# PICALM exerts a role in promoting CRC progression through ERK/MAPK signaling pathway

**DOI:** 10.1186/s12935-022-02577-z

**Published:** 2022-05-02

**Authors:** Xitao Zhang, Tianlai Liu, Jinlin Huang, Jianping He

**Affiliations:** 1 Department of Coloproctology, Zhujiang Hospital, Southern Medical University, 253 Gongye Middle Avenue, Haizhu, Guangzhou, 510280 Guangdong China; 2 Department of General Surgery, Shun De Hospital of Guang Zhou University of Chinese Medicine, 898 Jinsha Avenue, Shun De, Foshan, 510006 Guangdong China

**Keywords:** CRC, PICALM, Proliferation, Apoptosis, Migration, MAPK

## Abstract

**Background:**

Colorectal cancer (CRC) is a common malignant tumor in gastrointestinal tract with high incidence and mortality. In this study, the functions and potential mechanism of phosphatidylinositol-binding clathrin assembly protein (PICALM) in CRC were preliminarily explored.

**Methods:**

Based on the Cancer Genome Atlas database and immunohistochemistry staining, revealing that the expression level of PICALM in CRC tissues was higher than that in adjacent normal tissues.

**Results:**

Moreover, loss-of-function and gain-of-function assays in HCT 116 and RKO cells found that PICALM promotes proliferation and migration of CRC cells and inhibits apoptosis. Consistently, knockdown of PICALM inhibited tumorigenicity of CRC cells in vivo. Furthermore, Kyoto Encyclopedia of Genes and Genomes (KEGG) enrichment analysis showed that knockdown of PICALM resulted in the enrichment of MAPK signaling pathway. Treatment of CRC cells with MAPK inhibitor reversed the effects of PICALM overexpression on proliferation and apoptosis. In addition, overexpression of PICALM upregulated the protein levels of ERK1/2 (p-ERK1/2), MEK1/2 (p-MEK1/2), p38 (p-p38) and JNK (p-JNK), and these effects were partially alleviated by the treatment of MAPK inhibitor.

**Conclusions:**

In summary, the study presented the new discovery that PICALM promoted CRC progression through ERK/MAPK signaling pathway, which drew further interest regarding its clinical application as a promising therapeutic target.

**Supplementary Information:**

The online version contains supplementary material available at 10.1186/s12935-022-02577-z.

## Background

Colorectal cancer (CRC) is the common malignant tumor in gastrointestinal tract with high morbidity and mortality [1]. CRC affects 1.36 million individuals, accounting for about 10% of cancer patients worldwide [2]. The 5-year overall survival rate for CRC is estimated to be 64%, while for advanced metastatic CRC has dropped to nearly 12% [3]. In the case of adjuvant therapy, surgery alone or in combination with chemotherapy and radiotherapy is still the traditional treatment option for CRC [4]. In recent years, with the in-depth understanding of the mechanism of tumor development, the treatment of CRC has also made considerable progress [5, 6]. Currently, targeted drugs for CRC treatment mainly include anti-vascular endothelial growth factor (VEGF) (bevacizumab, aflibercept, ramucirumab), anti-epidermal growth factor receptor (EGFR) antibodies (cetuximab, panitumumab) and multi-kinase inhibitors (regorafenib) [7]. The combination of chemotherapy and the application of these new targeted drugs has significantly improved the overall survival of CRC patients [8]. Unfortunately, the effectiveness of some drugs can be altered by the rapid evolution of drug resistance and the occurrence of cancer recurrence [9, 10]. Therefore, the identification of novel molecule target to lay the theoretical foundation for reducing the severity of clinical CRC patients is imperative.

Phosphatidylinositol-binding clathrin assembly protein (PICALM) participates in clathrin-mediated endocytosis and autophagy, which is an important process in the intracellular transport of proteins, lipids, growth factors and neurotransmitters [11]. In addition, PICALM is required for the internalization and localization of cell-surface specific proteins such as transferrin receptor (TfR) and epidermal growth factor receptor (EGFR) [12–14]. PICALM loss leads to disruption in the internalization of TfR and EGFR, and PICALM overexpression has a major negative effect on the internalization of these receptors [15]. Moreover, PICALM was initially identified as a site of chromosomal translocation in a cell line derived from a lymphoma patient [16]. There is increasing evidence that alteration in PICALM is associated with several human diseases, such as leukaemia, Alzheimer's disease and Parkinson's disease [17–20]. However, whether PICALM reduction can accelerate cancer progression has not been analyzed, especially in CRC. To the best of our knowledge, our study is the first attempt to analyze the effects of PICALM in CRC.

## Methods

### Clinical sample collection

Tumor tissue (n = 80) and adjacent normal tissue samples (n = 80) of CRC patients were purchased from Shanghai Outdo Biotech Company (Shanghai, China). Permission for the collection and application of CRC samples was granted by the Ethics Committee of the Zhu Jiang Hospital of Southern Medical University. All obtained tissue specimens were immediately frozen in liquid nitrogen and stored at − 80 °C.

### Immunohistochemical (IHC) staining

Tumor specimens fixed with formalin were dewaxed with xylene for 15 min per time and hydrated with 100% ethanol. After repairing and blocking of the citrate antigen, the sample and PICALM antibody (1:50, Abcam, # ab172962) were incubated overnight in an incubator at 4 °C. After elution with PBS, secondary antibody IgG (1: 400, Abcam, # ab6721) was added, incubated at room temperature for 30 min, and washed with PBS. Tissue slices were first stained with DAB, and then with hematoxylin. The percentage of positively stained cells in the microscopic field was analyzed according to the German immunoreactivity score [21]. In brief, less than 25% scores 1; 25–50% scores 2; 50–75% scores 3; more than 75% scores 4. In addition, the staining intensity was graded as: 0 (no color), 1 (light yellow), 2 (light brown), or 3 (brown). For the purpose of statistical analysis for GSG2, the two grades were multiplied together and specimens were assigned to one of 4 levels: 0 score (−), 1–4 scores ( +), 5–8 scores (+ +), 9–12 scores (+ + +). The positive expression rate was expressed as the percent of the addition of (+ +) and (+ + +) to the total number. Notably, the high or low expression level of PICALM in tissues was defined by the median of IHC total scores calculated by positive cells and total staining intensity.

### Cells and reagents

The CRC cell lines HCT 116 and RKO (Cell Bank of the Chinese Academy of Sciences, Shanghai, China) were incubated in DMEM containing 10% fetal bovine serum (FBS) and placed at 37℃ with 5% CO_2_. Notably, all the cells were authenticated by short tandem repeat and confirmed negative for mycoplasma contamination prior to the experiments. In addition, MAPK inhibitor (FHPI, Selleck, USA, #SB202190) were used to treat cells in subsequent functional tests.

### Lentivirus transduction

Three RNA interference target sequences were designed using PICALM as template to construct lentivirus vector with RNA interference of the target gene. The sequences as follows: human-PICALM-1: 5’-TTGGATAAAAGTGGATTGCAA-3’, human-PICALM-2: 5’-TGCAGCATACAATGAAGGAAT-3’, human-PICALM-3: 5’-AACCTCATACCTCTTTAACAA-3’. Subsequently, the sequences were connected to lentivirus vector BR-V108 (5’-CCGGTTCTCCGAACGTGTCACGTTTCAAGAGAACGTGACACGTTCGGAGAATTTTTG-3’) using T4 DNA ligase (Thermo Scientific, USA, # EL0016) at enzyme digestion site (Age I, EcoR I). Next, the recombinant lentiviral (shCtrl and shPICALM) was transduced with HCT 116 and RKO cells (5 × 10^5^) using 5 μg lipofectamine 3000 (Invitrogen; Thermo Fisher Scientific, Inc.) in DMEM with 10% FBS at a multiplicity of infection of 10 for 30 min to generate. After cultured for 72 h at 37 °C, the expression of green fluorescent protein (GFP) was tested under the microscope to evaluate the transduction efficiency. Then, the stable cell lines were selected with puromycin as previously described [10]. Construction of recombinant lentivirus containing PICALM in subsequent experiments, for up-regulating PICALM expression, was also accomplished using similar methods as above.

### Quantitative real time PCR (qRT-PCR)

HCT 116 and RKO cell were collected and total RNA was extracted by Trizol. The concentration and quality of extracted RNA were determined by Nanodrop 2000/2000c spectrophotometer. The cDNA was obtained by reverse transcription using the Promega M-MLV kit. Finally, qRT-PCR was performed by using AceQ qPCR SYBR Green Master Mix (Vazyme, Nanjing, China). The primer sequences as follows, PICALM: sense, 5’-GAACCTTCCTGTTGCCAAACT and anti-sense, 5’-CTTAGTGGTTCCATTTCCGATG; GAPDH (internal reference): sense, 5’-TGACTTCAACAGCGACACCCA-3’ and anti-sense, 5’-CACCCTGTTGCTGTAGCCAAA-3’; The relative mRNA expression of PICALM was quantified with cycle threshold (Ct) values and normalized using the 2–∆∆Cq method.

### Western blot analysis

Total proteins of HCT 116 and RKO cell were extracted with 1 × cell lysis buffer (Promega, Madison, WI, USA) and protein concentrations were quantified by BCA protein detection kit (HyClone-Pierce, Waltham, MA, USA, # 23225). Subsequently, western blot analysis was performed by SDS-PAGE (10%) for constant pressure 80 V, 2 h. After proteins were transferred to polyvinylidene fluoride (PVDF) membrane, the blotted membranes were blocked with 5% non-fat milk in Tris-buffered saline for 1 h at room temperature and then incubated with primary antibodies (Additional file 2: Table S1) at 4 °C overnight. After washing with TBST, the blot was incubated with horseradish peroxidase (HRP) labeled polyclonal secondary antibody IgG (1:3000, Beyotime, Beijing, China, # A0208) at room temperature for 2 h. The protein signal bands were visualized using an enhanced chemiluminescence detection reagent (ECL and plus TM western blot system kit, Amersham, # RPN2232, Chalfont, UK).

### Cell proliferation assay

After HCT 116 and RKO cells of each group were digested with trypsin, the medium was completely resuspended in the cell suspension for counting, the cells were evenly spread at 2000 cells per well and continued culture. Next day, cell in each group were scanned and counted with Celigo (Nexcelom) and photographed under a fluorescence microscope. The cells were counted at the same time for 5 consecutive days and cell proliferation curves were drawn.

### Apoptotic analysis by flow cytometry

HCT 116 and RKO cells were cultured in 6-well plates at a density of 2000 per well for 7 days. After the cells were subsequently centrifuged, the cell pellet was washed with pre-chilled D-hanks and 1 × binding buffer at 4 °C (pH = 7.2 ~ 7.4). Cells were resuspended in 200 μL of 1 × binding buffer and stained with 10 μL of Annexin V-APC for 15 min at room temperature in the dark. Finally, 500 μL of 1 × binding buffer was added for detection by flow cytometry (Millipore, Guava easy Cyte HT).

### Transwell assay

HCT 116 and RKO cells were seeded in a 24-well plate at a density of 2000 per well and placed in the upper chamber of the Transwell. Meanwhile, the upper was added 100 μL serum-free medium and the lower chamber was added 600 μL of 30% FBS-containing medium. After 24 h, the upper chamber was upside down on absorbent paper to gently remove the medium and non-migrating cells. The cells were fixed with methanol for 30 min and soaked in 0.1% crystal violet staining solution for 20 min to stain the migrated cells. Finally, cells were observed at 200 × magnification under a microscope and photographed in five randomly selected fields of vision.

### Wound-healing assay

HCT 116 and RKO cells were inoculated into 96-well plate at a density of 3 × 10^4^ cells per well cultured for 24 h. The next day, cells were cultured in a medium containing 0.5% FBS for starvation treatment. The scratcher was gently pushed up from the center of the lower end of the 96-well plate to form a scratch and captured the images for 0 h. Subsequently, cells were placed in a 37 °C, 5% CO2 incubator for continued cultivation, and scanned with Cellomics (Thermo, ArrayScan VT1) at the 24 h, 30 h and 72 h. According to the migration area, the difference of cell healing ability was judged.

### Animal xenograft model

Animal research was approved by the Ethics Committee of Zhu Jiang Hospital of Southern Medical University and conducted in accordance with guidelines and protocols for animal care and protection. Adequate RKO cells were prepared and digested with trypsin and the cell suspension was resuspended in the complete medium (concentration of RKO cells suspension was set to 1E + 7 cells /mL). BALB/c female nude mice (4 weeks old) were purchased from Shanghai Jiesijie Experimental Animals Co., Ltd (Shanghai, China). Ten mice were randomly divided into two groups, shCtrl and shPICALM (n = 5). Each mouse was subcutaneously injected with 200 μL of tumor cells in the right forearm axilla. Tumor size and weight of mice were measured every other day until 15 days after subcutaneous injection. In the 55th day, the mice were anesthetized with 0.7% pentobarbital sodium intraperitoneally at a dose of 10 μL/g and the tumor load was evaluated under the IVIS spectral imaging system (emission wavelength 510 nm). Finally, after sacrificing the mice, tumors were removed, weighed, photographed and stored for further experiments.

### IHC for Ki67 staining

Tumor tissue was sectioned from the sacrificed mice. After tissue sections are repaired and blocked with citric acid antigen, antibody Ki67 (1: 200, Abcam, USA, # AB16667) was added to the shPICALM or shCtrl, respectively. Subsequently, mixed and incubated overnight at 4 °C. PBS elution for several times, secondary antibody IgG (1: 400, Abcam, USA, # AB6721) was added and incubated at room temperature for 30 min, PBS was washed again. Tissue slices were first stained with DAB, and then with hematoxylin. Images were collected with a photomicroscope and analyzed.

### Bioinformatics analysis

In order to reveal the impact of PICALM knockdown on downstream genes and signaling pathways, we used the Database for Annotation Visualization and Integrated Discovery (DAVID, https://david.ncifcrf.gov/) to analyze the Kyoto Encyclopedia of Genes and Genomes (KEGG).

### Statistical analysis

The results were representative of experiments repeated at least three times and the data was expressed as mean ± standard deviation (SD). Statistical was analyzed using GraphPad Prism 6.0 software (GraphPad Software Inc., San Diego, CA, USA) and SPSS 21.0 (IBM, Armonk, NY, USA). All tests were conducted using paired t test and one-way ANOVA followed by Bonferroni's post hoc test analysis. P values less than 0.05 were considered as statistically significant.

## Results

### PICALM expression is upregulated in CRC and correlates with prognosis of CRC patients

**Fig. 1:**
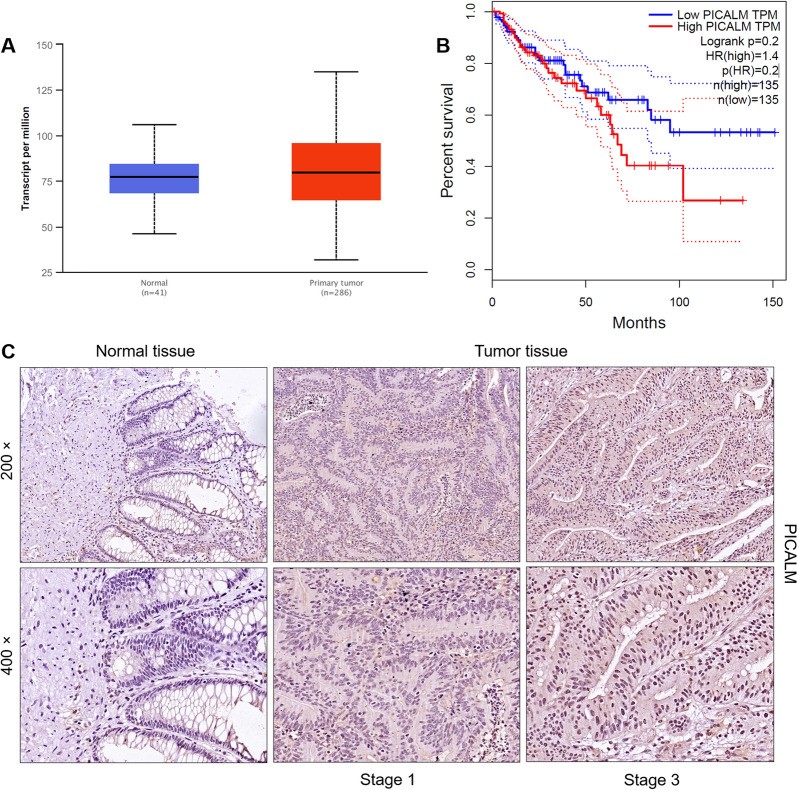
PICALM is highly expressed in CRC. **A** The mRNA level of PICALM in human CRC primary tumor (n = 286) and normal tissues (n = 41) was analyzed from the Cancer Genome Atlas database. **B** Correlation analysis between the expression level of PICALM and the survival time of CRC patients. **C** The expression of PICALM at the protein level was determined by IHC staining of CRC tissue (n = 80) and normal tissues (n = 80) (200 × and 400 × magnification)

Firstly, the mRNA level of PICALM in human CRC primary tumor (n = 286) and normal tissues (n = 41) was analyzed from public databases. Analysis of data from the Cancer Genome Atlas database showed that PICALM mRNA expression was upregulated in CRC compared with adjacent normal tissues (Fig. 1A). Data from this public database further showed that CRC patients with high levels of PICALM have shorter survival than those with low levels of PICALM (Fig. 1B). Then, we validated the expression of PICALM at the protein level by IHC staining of CRC tissue (n = 80) and normal tissues (n = 80), founding that the expression of PICALM in tumor tissues was significantly higher than that in normal tissues (Fig. 1A). Except for undetectable staining in some tissues, the proportion of CRC tissues (32/76) showing high PICALM expression was significantly higher than that of normal tissues (4/72) (P < 0.001) (Table 1). The Mann–Whitney U analysis was conducted based on the clinical case data, and the results showed the significant correlation between PICALM expression level and lymphatic metastasis (P = 0.042), stage (P = 0.013) of CRC patients (P = 0.015) (Table 2). Spearman correlation analysis confirmed that expression of PICALM was positively correlated with stage and lymphatic metastasis (Table 3). Collectively, PICALM was highly expressed in CRC and correlated with prognosis of CRC patients.

**Table 1:** Expression patterns in colorectal cancer tissues and para-carcinoma tissues revealed in immunohistochemistry analysis

PICALM expression	Tumor tissue		Para-carcinoma tissue		p value
	Cases	Percentage	Cases	Percentage	
Low	44	57.9	68	94.4	< 0.001
High	32	42.1	4	5.6

**Table 2:** Relationship between PICALM expression and tumor characteristics in patients with colorectal cancer

Features	No. of patients	PICALM expression		p value
		Low	High	
All patients	76	44	32	
Age (years)				0.112
< 65	34	16	18	
≥ 65	38	25	13	
Gender				0.903
Male	41	24	17	
Female	35	20	15	
Tumor size				0.330
< 5	36	23	13	
≥ 5	38	20	18	
Grade				0.222
2	66	40	26	
3	10	4	6	
Stage				0.013
1	7	7	0	
2	38	23	15	
3	27	13	14	
4	4	1	3	
T Infiltrate				0.508
T1	1	1	0	
T2	6	6	0	
T3	46	23	23	
T4	23	14	9	
Lymphatic metastasis (N)				0.042
N0	45	30	15	
N1	17	9	8	
N2	14	5	9	
Metastasis				0.174
M0	72	43	29	
M1	4	1	3

**Table 3:** Relationship between PICALM expression and tumor characteristics in patients with colorectal cancer

		PICALM
Lymphatic metastasis (N)	Spearman correlation coefficient	0.235
	Significance (two tails)	0.041
	N	76
Stage	Spearman correlation coefficient	0.287
	Significance (two tails)	0.012
	N	76

### PICALM is required for the cell proliferation and apoptosis of CRC

**Fig. 2:**
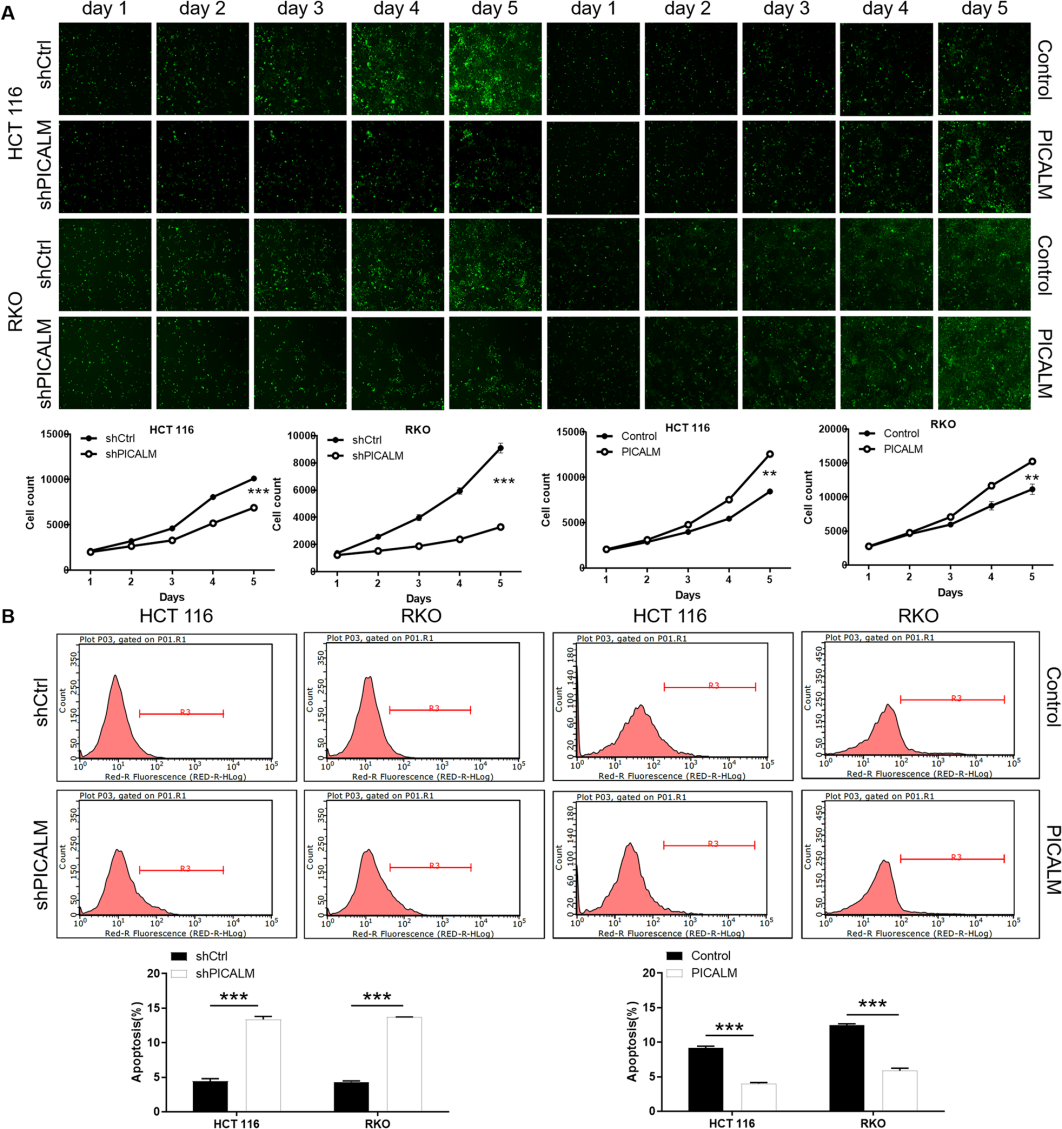
PICALM is required for CRC cell proliferation and apoptosis. **A** The effect of knockdown or overexpression of PICALM on HCT 116 and RKO cells proliferation was examined by cell counting experiment. **B** Flow cytometry analysis based on Annexin V-APC staining was utilized to detect the percentage of early apoptotic cell for HCT 116 and RKO cells. The representative images were selected from at least 3 independent experiments. The data was presented as the mean ± SD (n = 3). **P < 0.01, ***P < 0.001

To further identify the biological effects of PICALM in CRC, we constructed the knockdown of PICALM in CRC cell lines HCT 116 and RKO. Compared with the control group, the knockdown efficiency of PICALM in shPICALM-1/2/3 group was 43.1%, 78.0%, and 53.1%, respectively, (P < 0.001) (Additional file 1: Fig. S1A). As a result, shPICALM-2 was served as the most effective PICALM knockdown shRNA. The results of qRT-PCR showed that the mRNA levels of shPICALM group in HCT 116 and RKO cells were 55.2% and 88.6% lower than those in shCtrl group, respectively (P < 0.001) (Additional file 1: Fig. S1B). Western blot results indicated that the PICALM protein level in the shPICALM group was downregulated compared with shCtrl group (Additional file 1: Fig. S1C). Therefore, PICALM was downregulated by shRNA mediated knockdown in HCT 116 and RKO cells. Subsequently, the results of cell counting assays showed that the HCT 116 and RKO cells in shPICALM group both exhibited slower proliferation rate compared with shCtrl group (P < 0.001). In contrast, HCT 116 and RKO cells after PICALM overexpression showed enhanced proliferative capacity (P < 0.01) (Fig. 2A). Moreover, flow cytometry was applied to evaluate the percentage of apoptotic in HCT 116 and RKO cells with PICALM knockdown or overexpression. Downregulation of PICALM significantly enhanced the apoptosis rate of cells, while upregulation of PICALM attenuated the apoptosis of the cells (P < 0.001) (Fig. 2B). Taken together, PICALM played a key role in the proliferation and apoptosis of CRC cells.

### PICALM promotes the cells migration of CRC

**Fig. 3:**
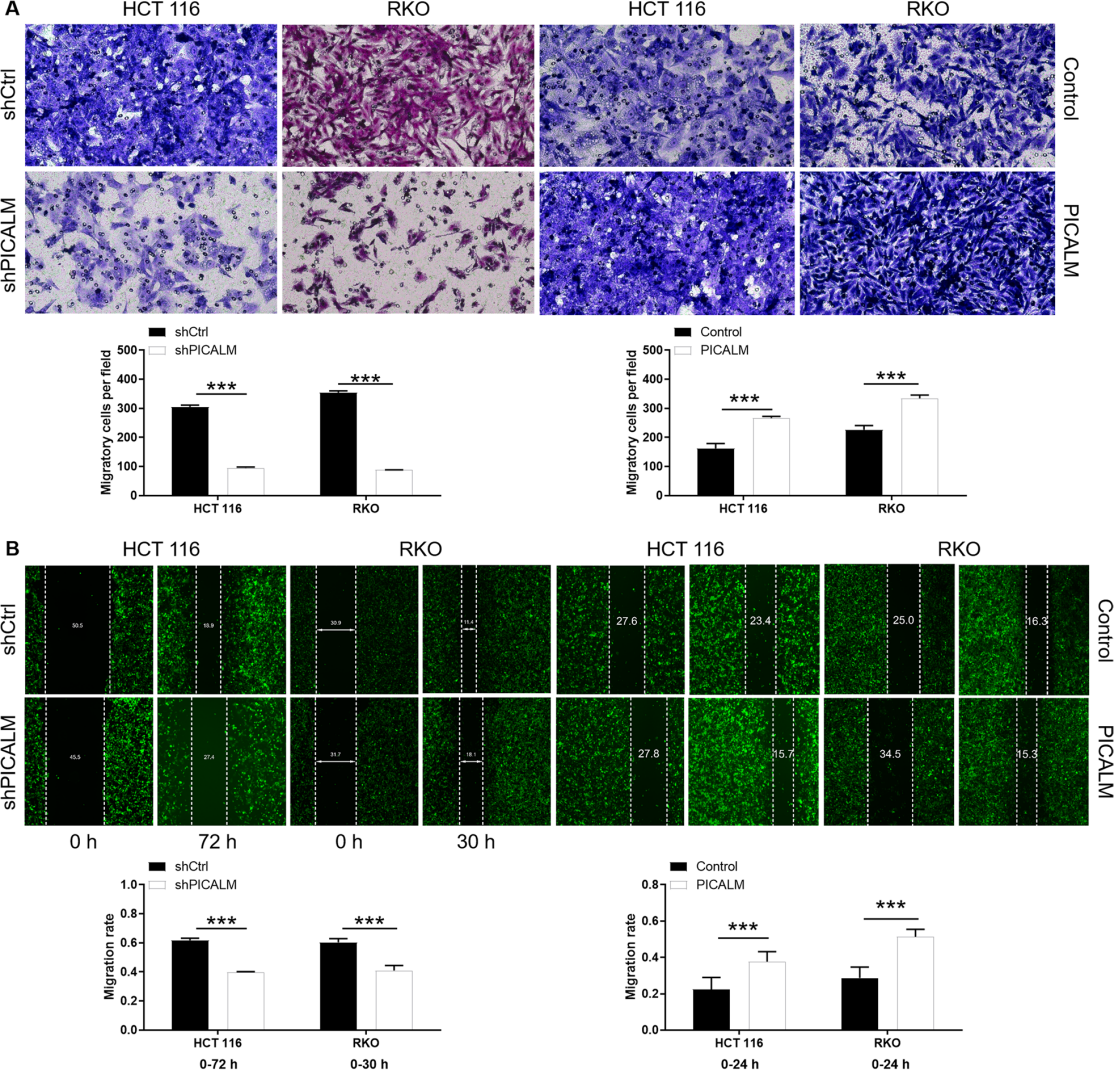
PICALM promotes CRC cell migration. The migratory capacity of PICALM knockdown or overexpressing HCT 116 and RKO cells was examined by Transwell assay (**A**) and wound-healing assay (**B**), respectively. The representative images were selected from at least 3 independent experiments. The data was presented as the mean ± SD (n = 3). ***P < 0.001

Cell migration capability was another character for malignant tumors. In this study, the effect of PICALM on cell migration was tested by Transwell assay and wound-healing assay. As illustrated in Fig. 3A, the Transwell assays demonstrated that PICALM knockdown reduced the migration of HCT 116 and RKO cells by 53% and 75%, respectively (P < 0.001). Meanwhile, overexpression of PICALM showed the exact opposite effect for the CRC cells migration (P < 0.001). Furthermore, wound-healing assays showed that the migration rate of HCT 116 and RKO cells in shPICALM group was 30% lower than that in shCtrl group (P < 0.001). As expected, PICALM-overexpressing CRC cells exhibit enhanced wound healing capacity (P < 0.001) (Fig. 3B). These results suggested that PICALM promoted the malignant progression of CRC tumors by regulating these cellular phenotypes of proliferation, apoptosis and migration.

### PICALM knockdown impairs the tumorigenic ability of CRC cells in vivo

**Fig. 4:**
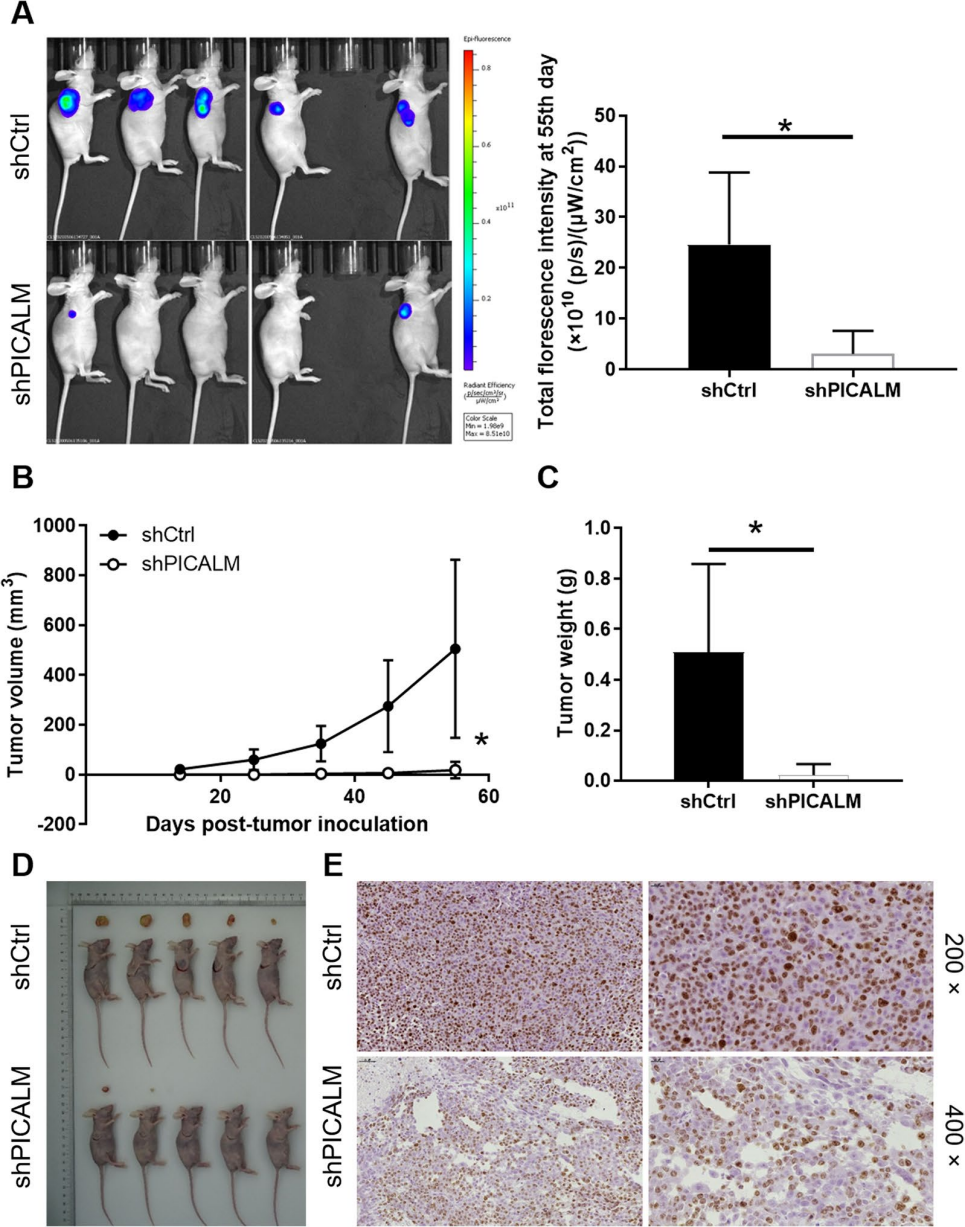
Knockdown of PICALM inhibits tumor growth in mice xenograft models. **A** The fluorescence intensity of tumors in the shCtrl group and shPICALM group was demonstrated. **B** The volume of tumors in shCtrl group and shPICALM group was measured after post-injection. **C** The average weight of tumors in shCtrl group and shPICALM group was measured. **D** Images of mice and tumors in shCtrl group and shPICALM group. **E** Tumor tissues in shCtrl group and shPICALM group were examined for Ki67 staining by IHC. The data was presented as the mean ± SD (n = 3). *P < 0.05

The mice xenograft models were established to further investigate the role of PICALM in the development of CRC. The results of fluorescence imaging showed that the fluorescence intensity of the shPICALM group was significantly lower than that of the shCtrl group, indicating that the tumor growth was slower after decreased the expression of PICALM (P < 0.05) (Fig. 4A). In addition, tumor growth was monitored for 55 days, the average tumor volume in the shPICALM group was only 18.44 mm^3^, which was significantly lower than in the control group (P < 0.05) (Fig. 4B). Moreover, the maximum tumor mass was only 0.1 g in the shPICALM group and 0.84 g in the shCtrl group. As expected, the mean tumor weight of the shPICALM group was 0.488 g less than that of the shCtrl group (P < 0.05) (Fig. 4C). As showed in Fig. 4D, we could intuitively find that tumor growth was significantly inhibited after PICALM knockdown. Additionally, Ki67 staining displayed that the proliferation index of tumor tissues in the shPICALM group was lower than that in the shCtrl group (Fig. 4E). Collectively, in vivo results confirmed that downregulation of PICALM played a role in tumorigenic damage in CRC.

### PICALM promotes CRC progression through the ERK/MAPK signaling pathway

**Fig. 5:**
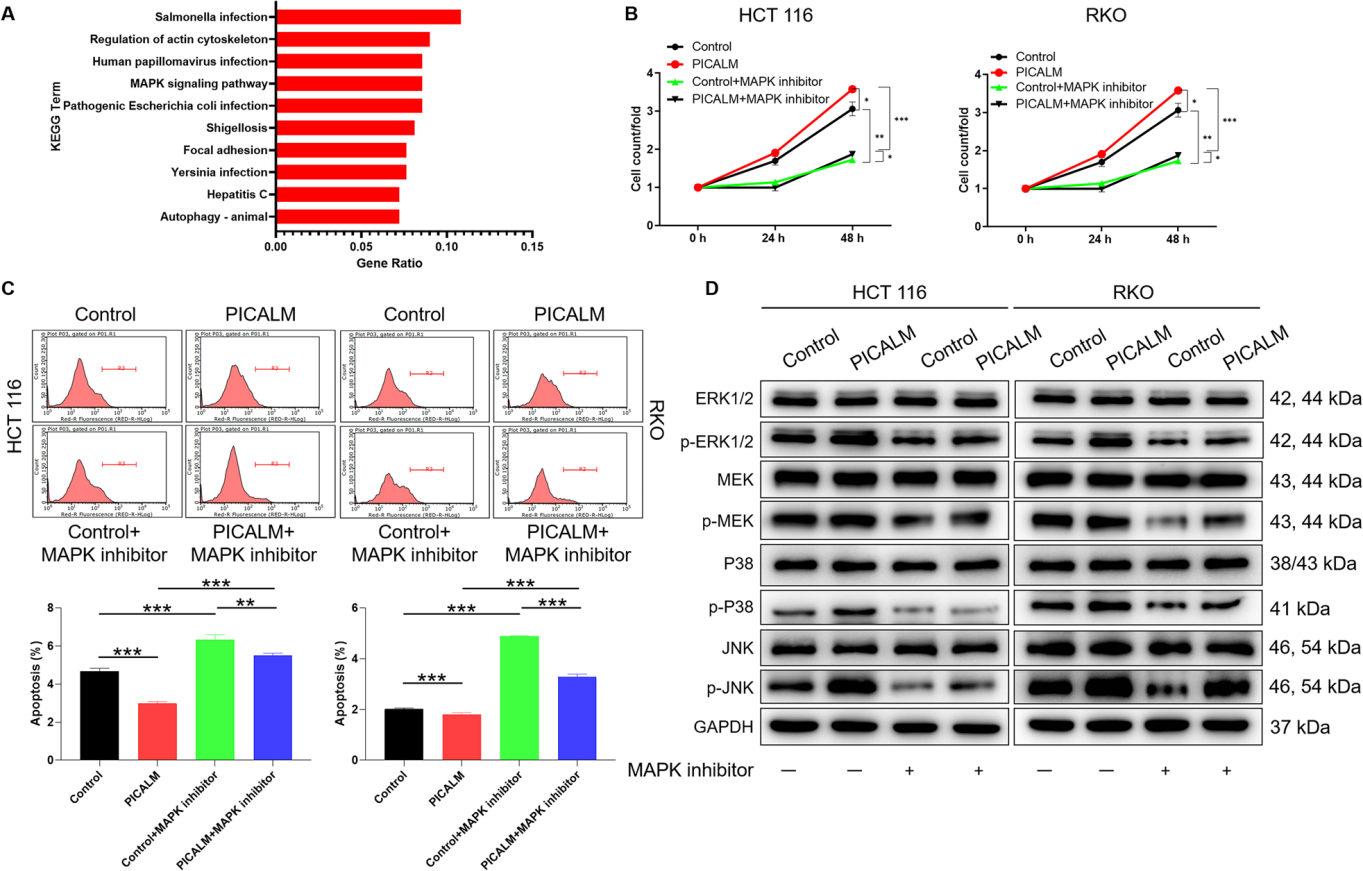
PICALM promotes CRC progression through the ERK/MAPK signaling pathway. **A** The potential mechanism of PICALM in CRC cells was analyzed by KEGG enrichment analysis. The names of enrichment pathways are shown on the left axis. The abscissa is enrichment factor. The high enrichment factor indicates that the enrichment of different proteins in this pathway is significant. **B** The effects of PICALM overexpression and MAPK inhibitor treatment on HCT 116 and RKO cells proliferation were detected by cell counting experiment. **C** The effects of PICALM overexpression and MAPK inhibitor treatment on HCT 116 and RKO cells apoptotic using flow cytometry analysis based on Annexin V-APC staining. **D** The effect of PICALM on the expression of canonical components of the ERK/MAPK signaling pathway was further examined by western blot analysis. The representative images were selected from at least 3 independent experiments. The data was presented as the mean ± SD (n = 3). *P < 0.05, **P < 0.01, ***P < 0.001

In order to explore the potential mechanism of PICALM in CRC, the KEGG was performed for enrichment analysis. As illustrated in Fig. 5A, PICALM knockdown resulted in a significant enrichment of the MAPK signaling pathway. Additionally, previous study reported that the activated MAPK pathway promoted the malignant progression of CRC [22, 23]. Thus, we inferred that PICALM may play a role in promoting CRC through the MAPK signaling pathway. Subsequently, HCT 116 and RKO cell lines with PICALM overexpression were treated with MAPK inhibitors (40 μM) to elucidate the role of MAPK in CRC. Notably, PICALM was the cells with PICALM overexpression and Control was negative control group. The results of cell count assays showed that the addition of MAPK inhibitor could reverse the promotion effect of PICALM overexpression on cells proliferation (P < 0.001) (Fig. 5B). Moreover, treatment with MAPK inhibitors weaken the inhibitory effect of PICALM overexpression on CRC cell apoptosis (P < 0.001) (Fig. 5C). The effect of PICALM on the expression of canonical components of the ERK/MAPK signaling pathway was further examined. The results indicated that overexpression of PICALM upregulated the protein levels of ERK1/2 (p-ERK1/2), MEK1/2 (p-MEK1/2), p38 (p-p38) and JNK (p-JNK), and these effects were partially alleviated by the addition of MAPK inhibitors (Fig. 5D). Taken together, PICALM may play a role in promoting CRC progress through ERK/MAPK signaling pathway.

## Discussion

CRC is a global concern in terms of morbidity and mortality. Surprisingly, approximately 90% of CRC deaths in CRC are associated with distant metastasis [24]. Therefore, with the in-depth understanding of the mechanisms of tumorigenesis and development is essential. In this study, the functions and potential mechanisms of PICALM in CRC have been preliminarily explored. Abnormal expression of PICALM has been shown to be associated with a variety of human diseases [17–20]. Not surprisingly, we identified that the expression level of PICALM was abnormally elevated in CRC. Moreover, the significant correlation between PICALM expression level and pathological grade of CRC patients was determined. Importantly, PICALM silencing could inhibit the malignant phenotypes of CRC tumor cells by inhibiting proliferation and migration as well as promoting apoptosis.

Furthermore, the potential mechanism of PICALM in CRC was analyzed by KEGG enrichment analysis. We found that PICALM knockdown resulted in significant enrichment of MAPK signaling pathway. The mitogen-activated protein kinase (MAPK) pathway has been reported to be involved in cell proliferation, differentiation, migration, senescence, and apoptosis [25]. Previous study reported that the activated MAPK pathway promoted the malignant progression of CRC [22, 23]. The present study demonstrated that the addition of MAPK inhibitor could slow down the effect of PICALM overexpression on proliferation and apoptosis of CRC cells. Additionally, extracellular signal-associated kinase (ERK1/2) is an important component of MAPK signaling pathway, which plays an important role in the progression of CRC [26]. Moreover, the effect of PICALM on the expression of canonical components of the ERK/MAPK signaling pathway was further examined. The results indicated that overexpression of PICALM upregulated the protein levels of ERK1/2 (p-ERK1/2), MEK1/2 (p-MEK1/2), p38 (p-p38) and JNK (p-JNK), and these effects were partially alleviated by the addition of MAPK inhibitors. Therefore, the results suggested that PICALM may play a role in promoting CRC progress through ERK/MAPK signaling pathway. The specific mechanism of how PICALM regulates CRC progression through ERK/MAPK signaling pathway remains to be further explored.

## Conclusion

The expression level of PICALM was abnormally elevated in CRC. In addition, PICALM was required for the proliferation, apoptosis and migration of CRC tumor cells. In summary, the study presented the new discovery that PICALM promoted CRC progression through ERK/MAPK, which provided a theoretical basis for the development of molecular drug targets for CRC.

## Supplementary Information

**Additional file 1: Figure S1.** Construction of a lentivirus-mediated PICALM knockdown CRC cell model. (A) Screening of efficient knockdown sequences targeting PICALM. (B-C) The specificity and validity of the lentivirus-mediated shRNA knockdown of PICALM expression was verified by qRT-PCR (C) and western blot analysis (D). The representative images were selected from at least 3 independent experiments. The data was presented as the mean ± SD (n = 3). ***P<0.001.

**Additional file 2: Table S1.** Antibodies used in western blot analysis.

## Data Availability

The datasets used and/or analysed during the current study are available from the corresponding author on reasonable request.
