# Supplementary material for: PICALM exerts a role in promoting CRC progression through ERK/MAPK signaling pathway
Source: Cancer Cell Int. 2022 May 2;22:178. doi: 10.1186/s12935-022-02577-z (PMC9063212; doi:10.1186/s12935-022-02577-z)
Supplement: Supplementary file 1 — Additional file 1: Figure S1. Construction of a lentivirus-mediated PICALM knockdown CRC cell model. (A) Screening of efficient knockdown sequences targeting PICALM. (B-C) The specificity and validity of the lentivirus-mediated shRNA knockdown of PICALM expression was verified by qRT-PCR (C) and western blot analysis (D). The representative images were selected from at least 3 independent experiments. The data was presented as the mean ± SD (n = 3). ***P<0.001. [file 12935_2022_2577_MOESM1_ESM.docx]

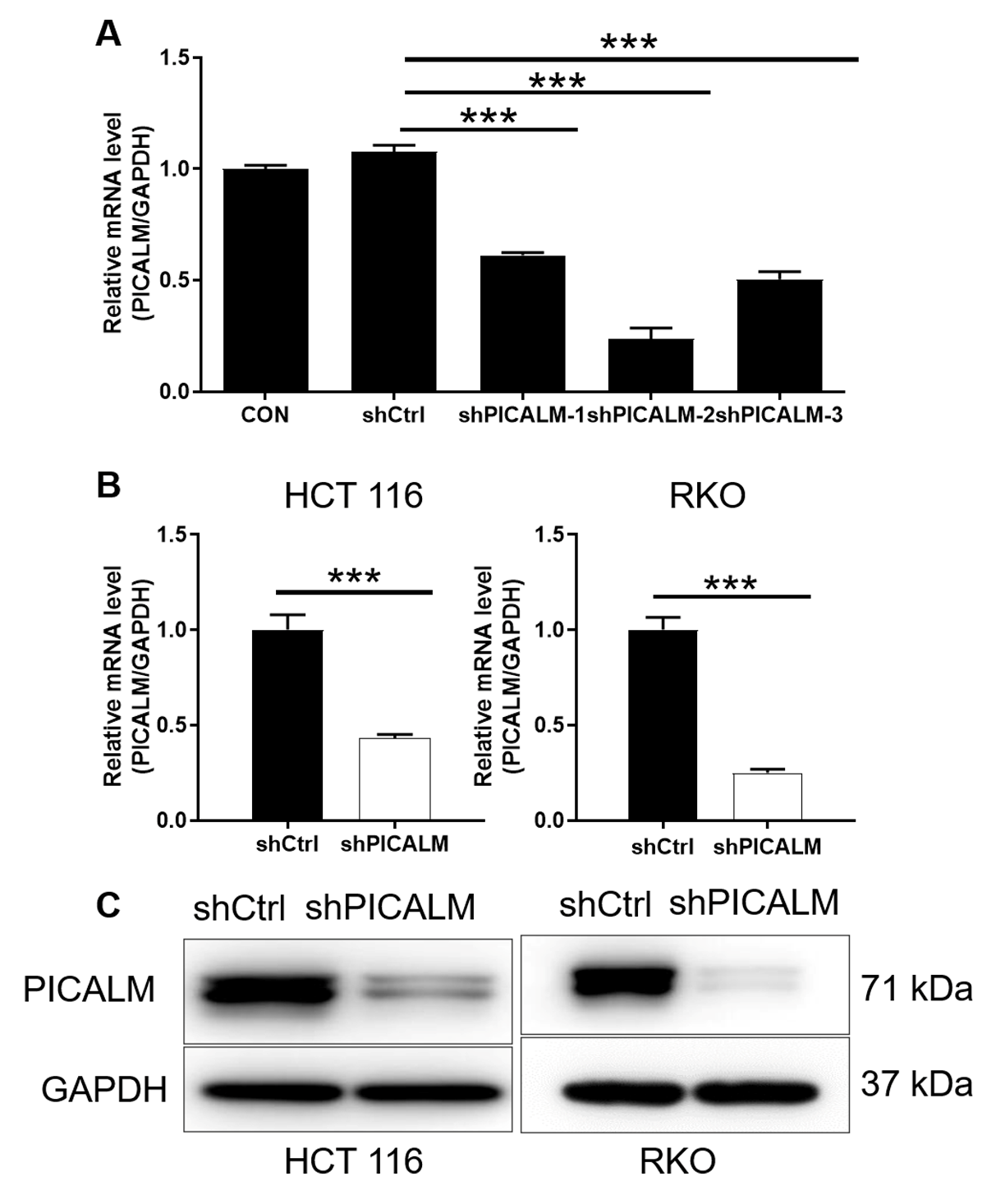


**Figure S1. Construction of a lentivirus-mediated PICALM knockdown CRC cell model.** (A) Screening of efficient knockdown sequences targeting PICALM. (B-C) The specificity and validity of the lentivirus-mediated shRNA knockdown of PICALM expression was verified by qRT-PCR (C) and western blot analysis (D). The representative images were selected from at least 3 independent experiments. The data was presented as the mean ± SD (n = 3). ***P<0.001.
