# Supplementary material for: PICALM exerts a role in promoting CRC progression through ERK/MAPK signaling pathway
Source: Cancer Cell Int. 2022 May 2;22:178. doi: 10.1186/s12935-022-02577-z (PMC9063212; doi:10.1186/s12935-022-02577-z)
Supplement: Supplementary file 2 — Additional file 2: Table S1. Antibodies used in western blot analysis. [file 12935_2022_2577_MOESM2_ESM.docx]

Table S1 Antibodies used in western blot analysis

| Primary antibodies | Band Size (KDa) | Dilution in WB | Source species | Company | Catalog No. |
| --- | --- | --- | --- | --- | --- |
| PICALM | 71 | 1:1000 | Rabbit | abcam | ab172962 |
| ERK1/2 | 42, 44 | 1:2000 | Rabbit | CST | 4695 |
| p-ERK1/2 | 42, 44 | 1:2000 | Rabbit | CST | 4695 |
| MEK | 43, 44 | 1:1000 | Rabbit | abcam | ab194754 |
| p-MEK | 43, 44 | 1:1000 | Rabbit | abcam | ab96379 |
| P38 | 38/43 | 1:1000 | Rabbit | abcam | ab170099 |
| p- P38 | 41 | 1:1000 | Rabbit | abcam | ab178867 |
| JNK | 46, 54 | 1:1000 | Mouse | CST | 3708 |
| p-JNK | 46, 54 | 1:2000 | Mouse | CST | 9255 |
| GAPDH | 37 | 1:3000 | Rabbit | Bioworld | AP0063 |
| HRP Goat Anti-Rabbit IgG (WB) |  | 1:3000 |  | Beyotime | A0208 |
| HRP Goat Anti-Mouse IgG (WB) |  | 1:200 |  | Beyotime | A0216 |
